# Synthesis of 2′-Fluoro RNA by Syn5 RNA polymerase

**DOI:** 10.1093/nar/gkv367

**Published:** 2015-04-20

**Authors:** Bin Zhu, Alfredo Hernandez, Min Tan, Jan Wollenhaupt, Stanley Tabor, Charles C. Richardson

**Affiliations:** 1 Department of Biological Chemistry and Molecular Pharmacology, Harvard Medical School, Boston, MA 02115, USA; 2 Departments of Medicine, Brigham and Women's Hospital and Harvard Medical School, Boston, MA 02115, USA; 3 Institute of Chemistry and Biochemistry, Free University Berlin, Berlin 14195, Germany

## Abstract

The substitution of 2′-fluoro for 2′-hydroxyl moieties in RNA substantially improves the stability of RNA. RNA stability is a major issue in RNA research and applications involving RNA. We report that the RNA polymerase from the marine cyanophage Syn5 has an intrinsic low discrimination against the incorporation of 2′-fluoro dNMPs during transcription elongation. The presence of both magnesium and manganese ions at high concentrations further reduce this discrimination without decreasing the efficiency of incorporation. We have constructed a Syn5 RNA polymerase in which tyrosine 564 is replaced with phenylalanine (Y564F) that further decreases the discrimination against 2′-fluoro-dNTPs during RNA synthesis. Sequence elements in DNA templates that affect the yield of RNA and incorporation of 2′-fluoro-dNMPs by Syn5 RNA polymerase have been identified.

## INTRODUCTION

A substitution of 2′-fluoro (2′-F) for the 2′-hydroxyl group in RNA does not substantially change the conformation of RNA but substantially increases its melting temperature, chemical stability and resistance to ribonuclease. These properties result in a longer survival time of 2′-F RNAs (or 2′-F DNAs if all the 2′ positions bear 2′-fluoro groups) *in vitro* and *in vivo*. 2′-F RNAs are used widely in studies of ribozymes, the selection of aptamers and in RNA interference (1–8).

The most common 2′-F substitutions used in RNAs are 2′-F-dCMP and 2′-F-dUMP. RNAs containing these substitutions are resistant to RNase A, the most common RNase contaminant, which recognizes the 2′-OH of pyrimidines for cleavage (9). 2′-F RNA can be synthesized enzymatically (10–12), and currently the standard enzyme for the preparation of 2′-F RNA is the bacteriophage T7 RNA polymerase Y639F in which tyrosine 639 is replaced with phenylalanine (13), commercialized by Epicentre as T7 R&DNA™ Polymerase and The DuraScribe® T7 Transcription Kit. The Y639F alteration in T7 RNA polymerase greatly reduces discrimination between non-canonical and canonical nucleoside triphosphates. However such discrimination is still substantial, especially when multiple 2′-modified NTPs or 2′-modified GTP (the strict initiation nucleotide for T7 RNA polymerase) are included in the reaction (13,14).

Recently we characterized a single-subunit RNA polymerase from marine cyanophage Syn5 (15) and described some of its advantages as a tool for *in vitro* transcription. These advantages include product-3′-homogeneity, high processivity, flexible initiating nucleotide and tolerance to salt (16). In the current study we have investigated the ability of wild-type and altered Syn5 RNA polymerases to synthesize RNA containing 2′-F moieties. We have found that wild-type Syn5 RNA polymerase has an inherent low discrimination against 2′-F-dNTPs compared to T7 RNA polymerase. Syn5 RNA polymerase also retains high activity in the presence of manganese ions, which further decrease its discrimination against 2′-F-dNTPs. A single amino acid change in Syn5 RNA polymerase further reduces its ability to discriminate against 2′-F-dCTP and 2′-F-dUTP. We also describe an improved expression system for the overproduction of Syn5 RNA polymerase in *Escherichia coli*, characterize sequence elements in DNA templates that reduce the yield of RNA synthesized by Syn5 RNA polymerase, and describe ways to improve the yield in the presence of these DNA sequences.

## MATERIALS AND METHODS

### Materials

Oligonucleotides were obtained from Integrated DNA Technology (oligonucleotides less than 60 nt were ordered at the 25 nmole scale and those longer than 60 nt were ordered at the 4 nmole Ultramer scale). DNA purification kits and Ni-NTA resin were from Qiagen. Preparative Superdex 200 for gel filtration was from GE Healthcare. Restriction endonucleases, T4 DNA ligase, Vent_R_^®^ DNA Polymerase, Q5^®^ Site-Directed Mutagenesis Kit, T7 RNA polymerase and *E. coli* inorganic pyrophosphatase were from New England Biolabs. T7 R&DNA™ Polymerase (T7-Y639F RNA polymerase) was from Epicentre. DNA Clean & Concentrator™-5 kit was from ZYMO Research. RNaseOUT™ recombinant ribonuclease inhibitor was from Invitrogen. NTPs were from USB and 2′-F-dNTPs were from Trilink.

### Protein expression and purification

We have modified the original expression vector (15,16) to improve the expression of His-tagged Syn5 RNA polymerase by removing the internal Syn5 promoter sequence within the Syn5 RNA polymerase gene without changing the encoded amino acid (Supplementary Figure S1A). With this vector (Syn5 RNAP-NP-pET24) the synthesized Syn5 RNA polymerase will not initiate transcription from the internal promoter sequence, an event that would deplete the rNTP pools and inhibit the synthesis of the full-length mRNA for Syn5 RNA polymerase. *E. coli* cells harboring this vector grow significantly faster than those carrying the original plasmid and contain higher levels of overproduced protein (Supplementary Figure S1B and C). Y564F and Y574F mutations were introduced into the Syn5 RNA polymerase gene in the Syn5 RNAP-NP-pET24 vector by polymerase-chain-reaction mutagenesis.

The purification procedure was modified from that previously described (16). *E. coli* BL21(DE3) cells containing the plasmid Syn5 RNAP-NP-pET24 were grown in 2 L of LB medium containing 50 μg/ml kanamycin at 37°C until they reached an OD_600_ of 1.2. The gene for Syn5 RNA polymerase was induced by the addition of 0.5 mM IPTG at 25°C and incubation was continued for 4–8 h. The cells were harvested, resuspended in 50 mM sodium phosphate, pH 8.0, 100 mM NaCl, and lysed by three cycles of freeze-thaw in the presence of 0.5 mg/ml lysozyme. Solid NaCl was added to the lysed cells to a final concentration of 2 M and then the cleared lysate was collected after centrifugation. 5 ml Ni-NTA agarose was added to the clear lysate and gently mixed at 4°C overnight. The resin was loaded and collected in a column and washed with 60 ml of Wash Buffer (50 mM sodium phosphate, pH 8.0, 2 M NaCl and 10 mM imidazole). Syn5 RNA polymerase was eluted from the column using 30 ml Elution Buffer (50 mM sodium phosphate, pH 8.0, 2 M NaCl and 100 mM imidazole). Eluted protein was concentrated to 1 ml using an Amicon Ultra-15 Centrifugal Filter Units (Millipore). The concentrated sample was loaded directly onto a 200 ml preparative Superdex 200 column. The gel filtration buffer contained 20 mM Tris-HCl pH 7.5, 2 M NaCl, 0.5 mM DTT and 0.5 mM EDTA. Fractions were analyzed on SDS-PAGE gels and those fractions that contained homogenous Syn5 RNAP were pooled. The pooled fractions were concentrated by Amicon Ultra-15 Centrifugal Filter followed by dialysis against Dilution Buffer (50 mM Tris-HCl pH 8.0, 100 mM NaCl, 20 mM β-ME, 1 mM EDTA, 50% glycerol and 0.1% Triton® X-100) and stored at −20°C. Dilutions for enzyme assays were carried out using Dilution Buffer. The yield of Syn5 RNA polymerase following this procedure was greater than 20 mg protein per gram of wet cells, with the majority of the soluble Syn5 RNA polymerase (>80%) retained in the flow-through and wash fractions of the Ni-NTA step. Repetition of this procedure using the flow-through fraction of the Ni-NTA column generates similar amounts of purified protein. Syn5 RNA polymerase mutants Y564F and Y574F were purified following the same procedure resulting in a similar yield.

### DNA templates for transcription assays

DNA templates were constructed by annealing the complementary synthesized oligonucleotides listed below. Only the non-template strands are shown, listed 5′-3′, with the promoters shown in bold and the nucleotides corresponding to residues 1–12 of the RNA products underlined.

**T1**: GGT**ATTGGGCACCCGTAA**GGAGAACCTTAA GGTTTAACTTTAAGACCCTTAAGTG
**T2**: GCC**ATTGGGCACCCGTAA**GGGAGAGAGCAT CGCTTGGTGCAGATCGGGAC
**T3** (template for human mitochondrial tRNA^Pro^_UGG_): GCC**ATTGGGCACCCGTAA**CAGAGAATAGTT TAAATTAGAATCTTAGCTTTGGGTGCTAATGGTGGAGTTAAAGACTTTTTCTCTGACCA
**T4**: GGT**ATTGGGCACCCGTAA**GGAGAAGAAGAA GGTTTAACTTTAAGACCCTTAAGTG
**T5**: GGT**ATTGGGCACCCGTAA**GGGGAAGAAGAA GGTTTAACTTTAAGACCCTTAAGTG
**T6**: GGT**ATTGGGCACCCGTAA**GAAGAAGAAGAA GGTTTAACTTTAAGACCCTTAAGTG
**T7**: GGT**ATTGGGCACCCGTAA**AGAGAAGAAGAA GGTTTAACTTTAAGACCCTTAAGTG
**T8**: GGT**ATTGGGCACCCGTAA**GCAGAAGAAGAA GGTTTAACTTTAAGACCCTTAAGTG
**T9**: GGT**ATTGGGCACCCGTAA**GGAAAAGAAGAA GGTTTAACTTTAAGACCCTTAAGTG
**T10**: GGT**ATTGGGCACCCGTAA**GGATTTGAAGAA GGTTTAACTTTAAGACCCTTAAGTG
**T11**: GGT**ATTGGGCACCCGTAA**GGACCCGAAGAA GGTTTAACTTTAAGACCCTTAAGTG
**T12**: GGT**ATTGGGCACCCGTAA**GGAGAAAAAGAA GGTTTAACTTTAAGACCCTTAAGTG
**T13**: GGT**ATTGGGCACCCGTAA**GGAGAAGCGGAA GGTTTAACTTTAAGACCCTTAAGTG
**T14**: GGT**ATTGGGCACCCGTAA**GGAGAATTTGAA GGTTTAACTTTAAGACCCTTAAGTG
**T15**: GGT**ATTGGGCACCCGTAA**GGAGAACCCGAA GGTTTAACTTTAAGACCCTTAAGTG
**T16**: GGT**ATTGGGCACCCGTAA**GGAGAAGAAAAA GGTTTAACTTTAAGACCCTTAAGTG
**T17**: GGT**ATTGGGCACCCGTAA**GGAGAAGAATTT GGTTTAACTTTAAGACCCTTAAGTG
**T18**: GGT**ATTGGGCACCCGTAA**GGAGAAGAACCC GGTTTAACTTTAAGACCCTTAAGTG
**T19**: GGT**ATTGGGCACCCGTAA**GGAGTAGAAGAA GGTTTAACTTTAAGACCCTTAAGTG
**T20**: GGT**ATTGGGCACCCGTAA**GGAGTAGTAGAA GGTTTAACTTTAAGACCCTTAAGTG
**T21**: GGT**ATTGGGCACCCGTAA**GGAGTTGAAGAA GGTTTAACTTTAAGACCCTTAAGTG
**T22**: GGT**ATTGGGCACCCGTAA**GCAGAACCTTAA GGTTTAACTTTAAGACCCTTAAGTG
**T23**: GGT**ATTGGGCACCCGTAA**CAGGAACCTTAA GGTTTAACTTTAAGACCCTTAAGTG
**T24**: GGT**TAATACGACTCACTATA**GGAGAACCTT AAGGTTTAACTTTAAGACCCTTAAGTG
**T25**: GGT**TAATACGACTCACTATA**GGGGAACCTT AAGGTTTAACTTTAAGACCCTTAAGTG
**T26**: GGT**TAATACGACTCACTATA**GCAGAACCTT AAGGTTTAACTTTAAGACCCTTAAGTG
**T27**: GGT**TAATACGACTCACTATA**CAGGAACCTT AAGGTTTAACTTTAAGACCCTTAAGTG
**T28**: GGT**TAATACGACTCACTATA**GGAGAAGAAG AAGGTTTAACTTTAAGACCCTTAAGTG
**T29**: TCC**ATTGGGCACCCGTAA**GCAGGGAGGACG ATGCGGGCCTTCGTTTGTTTCGTCCACAGACGACTCGCCCGA
**T32**: GGT**ATTGGGCACCCGTAA**UGAGAACCTTAA GGTTTAACTTTAAGACCCTTAAGTG
**T33**: **ATTGGGCACCCGTAA**GGGAGGACGATGCGGGCCTTCGTTTGTTTCGTCCACAGACGACTCGCCCGA

Plasmid pUC19 with a single Syn5 promoter and three guanosine residues (5′-**ATTGGGCACCCGTAA**GGG-3′) inserted between the BamHI and XbaI sites (16) was linearized by NdeI to serve as template **T30**, which results in a 2700 nt RNA. The first nine nucleotides of the RNA generated from this template are GGGUCUAGA. The linearized plasmid template **T31** was derived from template T30 that was modified so that the first nine nucleotides of the RNA generated from this template are GCAGAAGAA. Linearized plasmids were purified with DNA Clean & Concentrator™-5 kit (ZYMO Research) prior to use in transcription assays.

### Transcription assays

**Figure 1.**
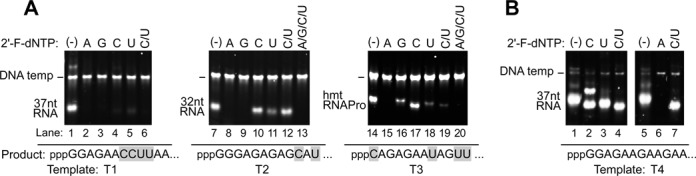
2′-F-dNMP incorporation into transcripts synthesized by Syn5 RNA polymerase on various DNA templates. Products of transcription reactions were separated by native gel electrophoresis and then stained with ethidium bromide. **(A)** Efficiency of 2′-F-dNMP incorporation by Syn5 RNA polymerase is correlated to the sequence at the 5′ end of the RNA. Transcription reactions were carried out using Syn5 RNA polymerase on templates T1 (lanes 1–6), T2 (lanes 7–13) and T3 (lanes 14–20). In some reactions some of the four NTPs were replaced by the corresponding 2′-F analogs, as indicated at the top of the gel. The first 12 nucleotides at the 5′-end of the transcripts produced on each template are shown below each gel. The position of the migration of the DNA templates and the RNA products are marked on the left of the gel. **(B)** Efficiency of 2′-F-dNMP incorporation by Syn5 RNA polymerase on template T4. Normal NTPs were replaced by the corresponding 2′-F analogs as indicated at the top of each lane. The first 12 nucleotides at the 5′-end of the transcripts produced by template T4 are shown below each gel. The position of the migration of the DNA templates and the RNA products are marked on the left of the gel.

**Figure 2.**
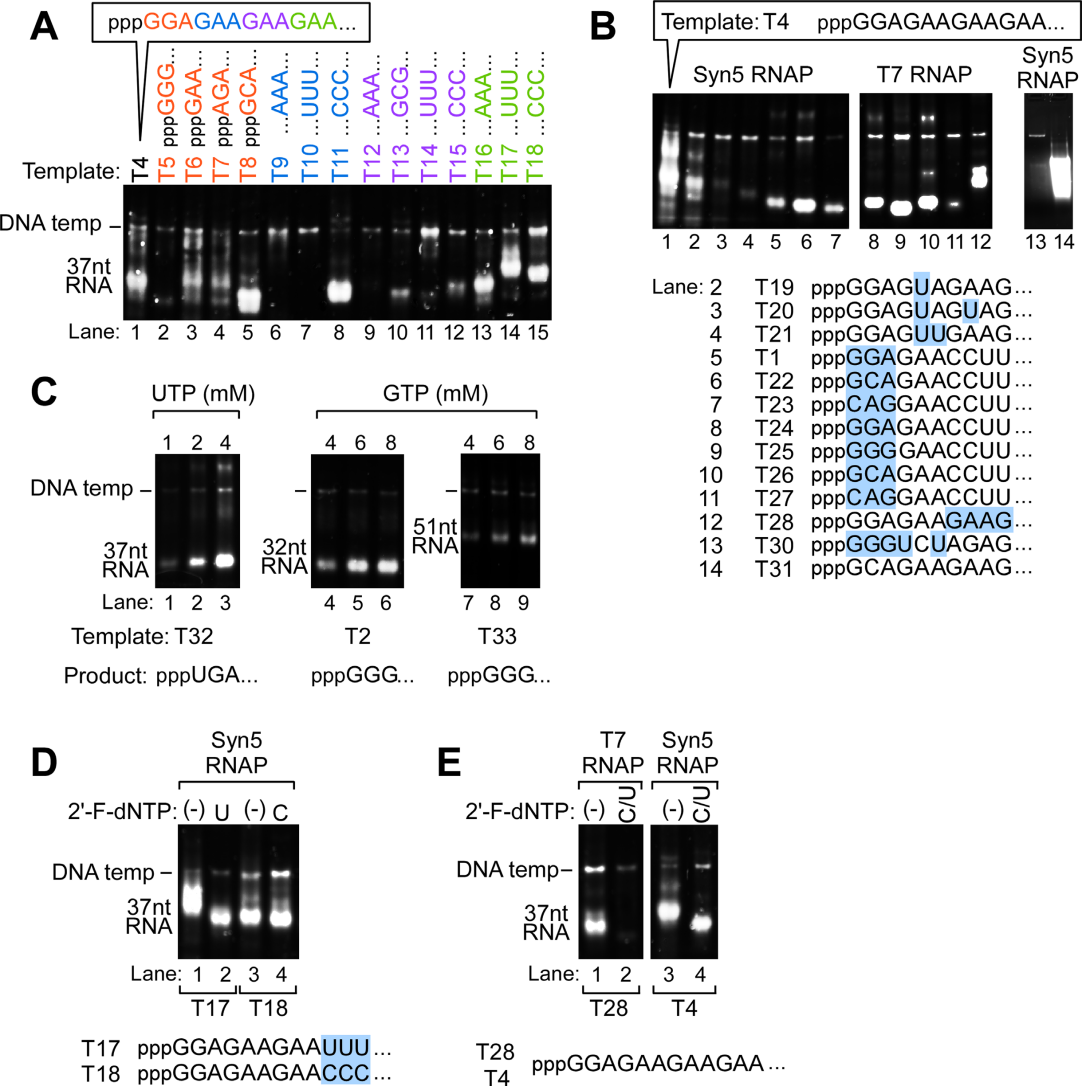
Impact of initial sequence of transcript on the yield of transcripts synthesized by Syn5 RNA polymerase. **(A)** Influence of different sequences at various positions in the first 12 nucleotides at the 5′ end of transcripts on the yield of products synthesized by Syn5 RNA polymerase. The DNA template used in each reaction is indicated at the top of each lane. All of the templates were derived from template T4, from which the first 12 nucleotides at the 5′ end of the RNA product are shown at the top of the gel. The variations in the sequence of RNA products from template T4 synthesized on each template are color coded; variations in the first three nucleotides are in orange, nucleotides 4–6 in blue, nucleotides 7-9 in purple and nucleotides 10–12 in green. The position of the migration of the DNA templates and the RNA products are marked on the left of the gel. **(B)** Influence of different sequences at various positions in the first 10 nucleotides at the 5′ end of transcripts on the yield of products synthesized by Syn5 and T7 RNA polymerases. Transcription reactions were carried out using Syn5 RNA polymerase (lanes 1–7, 13 and 14) and T7 RNA polymerase (lanes 8–12). The templates used for each reaction and the sequence of the first 10 nucleotides at the 5′ end of each RNA are shown at the bottom of the gel. Variations in RNA products encoded by each template are in blue background. **(C)** Influence of NTP concentration on the yield of products synthesized by Syn5 RNA polymerase. Transcription reactions were carried out on templates T32 (lanes 1–3), T2 (lanes 4–6) and T33 (lanes 7–9). The first three nucleotides of the transcript synthesized on each of these templates are UGA (T32), GGG (T2) and GGG (T33), as indicated at the bottom of the gel. The concentration of the NTP being varied in each reaction mixture is shown at the top; the concentrations of the other three NTPs were fixed at 4 mM. The position of the migration of the DNA templates and the RNA products are marked on the left of the gel. **(D)** and **(E)** Incorporation of 2′-F-dCMP and/or 2′-F-dUMP into transcripts synthesized by Syn5 and T7 RNA polymerases. The templates used for each reaction (T18, T17, T28 and T4) and the sequence of the first 12 nucleotides at the 5′ end of each RNA are shown at the bottom of the gel. The NTP analog present in each reaction is indicated at the top of the gel where ‘C/U’ corresponds to reactions carried out in the presence of both 2′-F-dCTP and 2′-F-dUTP.

The reaction conditions we used initially were those most commonly used for *in vitro* transcription reactions using T7 RNA polymerase. The reaction mixtures were analyzed on gels stained with ethidium bromide, which allowed for a direct comparison between the relative amount of RNA products and DNA templates. Reaction mixtures (10 μl) contained 40 mM Tris-HCl pH 8.0, 2 mM spermidine, 20 mM DTT, 20 mM MgCl_2_ (or MnCl_2_, or both, as specified), 4 mM of each of the 4 rNTPs (with 0 to 4 of the rNTPs replaced by their 2′-F-dNTP analogs), 1.5 U/μl RNaseOUT^TM^ recombinant ribonuclease inhibitor, 0.006 U/μl *E. coli* inorganic pyrophosphatase, DNA template (2 μM annealed oligonucleotides (∼200 ng/μl) or 20 nM linearized 2700 bp plasmid (∼35 ng/μl) and RNA polymerase (4 μM Syn5 RNAP polymerase or its Y564F mutant, or 5 U/μl T7 RNA polymerase from New England Biolabs or 5 U/μl T7-Y639F RNA polymerase (T7 R&DNA™ Polymerase) from Epicentre). Reaction mixtures were incubated at 37°C for 4 h (8 h for assays described in Figures 5 and 6 lower gels). 1 μl of the mixtures were mixed with 7 μl H_2_O and 7 μl denaturing RNA loading dye (New England Biolabs). Samples (5 μl was used for loading onto all gels except those shown in Figures 1B and 2A, which used 2.5 μl) were then loaded onto 10% TBE native gels (Bio-Rad) or 2% TAE agarose gels (for the 2700 nt RNA). DNA templates and RNA products were separated by electrophoresis and visualized by staining with ethidium bromide. Transcription yield was calculated based on comparison of the intensity of the product band to that of the DNA template band (Supplementary Table S1). To confirm the yield of some reactions, one unit of DNase I (New England Biolabs) was added to each reaction mixture and incubated for an additional 20 min at 37°C to remove the DNA templates. The transcripts were then purified serially with phenol/chloroform extraction, Micro Bio-Spin™ P-30 Gel Columns (RNase-free, Bio-Rad) and ethanol precipitation. The amount of purified transcript was determined using NanoPhotometer® (Implen).

**Figure 3.**
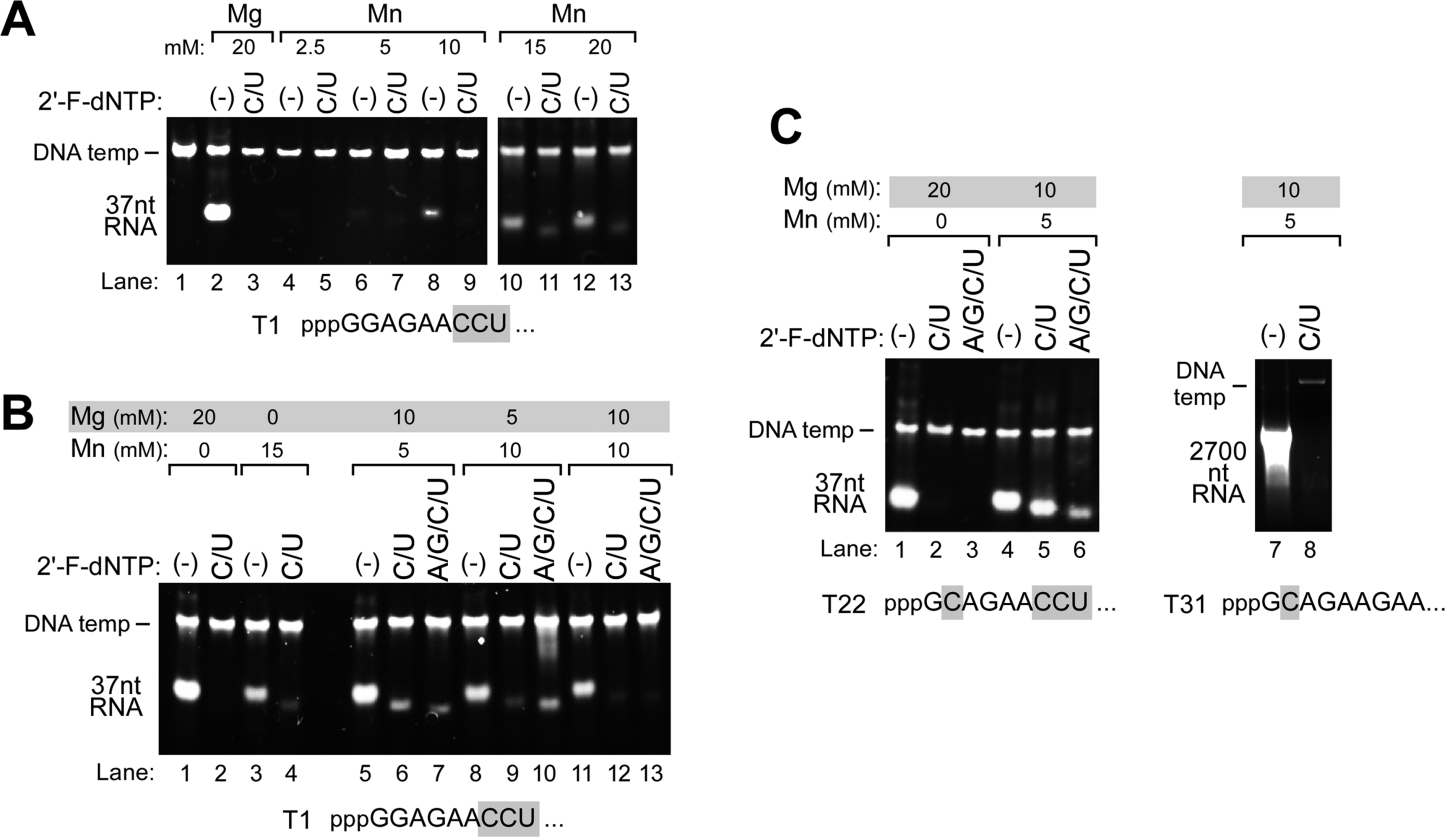
Effect of Mn^2+^ ions on 2′-F-dNMP incorporation by Syn5 RNA polymerase. Products of transcription reactions were separated by native gel electrophoresis and then stained with ethidium bromide**. (A)** Incorporation of 2′-F-dCMP and 2′-F-dUMP by Syn5 RNA polymerase in the presence of Mg^2+^ or Mn^2+^. Transcription reactions were carried out by Syn5 RNA polymerase on the template T1; the sequence of the first nine nucleotides of the 37 nt transcript produced on this template is shown at the bottom of the figure, with the C and U residues in gray background. The metal ion present in each reaction and its concentration are shown at the top of the gel. The reaction mixtures that contained both 2′-F-dCTP and 2′-F-dUTP (C/U) are also indicated. Lanes 1 contains only the DNA template as a marker. The position of the migration of the DNA templates and the RNA products are marked on the left of the gel. **(B)** Incorporation of 2′-F-dNMPs by Syn5 RNA polymerase in the presence of a mixture of Mg^2+^ and Mn^2+^ ions. Transcription reactions were carried out by Syn5 RNA polymerase on the template T1; the sequence of the first nine nucleotides of the 37 nt transcript produced on this template is shown at the bottom of the gel, with the C and U residues in gray background. **(C)** Incorporation of 2′-F-dNMPs into a 37 nt transcript and a 2700 nt transcript by Syn5 RNA polymerase in the presence of a mixture of Mg^2+^ and Mn^2+^. Transcription reactions were carried out using either template T22, which encodes a 37 nt transcript (lanes 1–6), or T31, which encodes a 2700 nt transcript (lanes 7 and 8). The sequence of the first nine nucleotides of each transcript is shown at the bottom of the gel, with the C and U residues in gray background. The metal ions present in each reaction and their concentrations are shown at the top of the gel; a mixture of 10 mM Mg^2+^ and 5 mM Mn^2+^ were present in the reaction mixtures in lanes 4–8. Also the reaction mixtures that contained both 2′-F-dCTP and 2′-F-dUTP or all four 2′-F-dNTPs are shown at the top of the gel. The position of the migration of the DNA templates and the RNA products are marked on the left of the gel.

**Figure 4.**
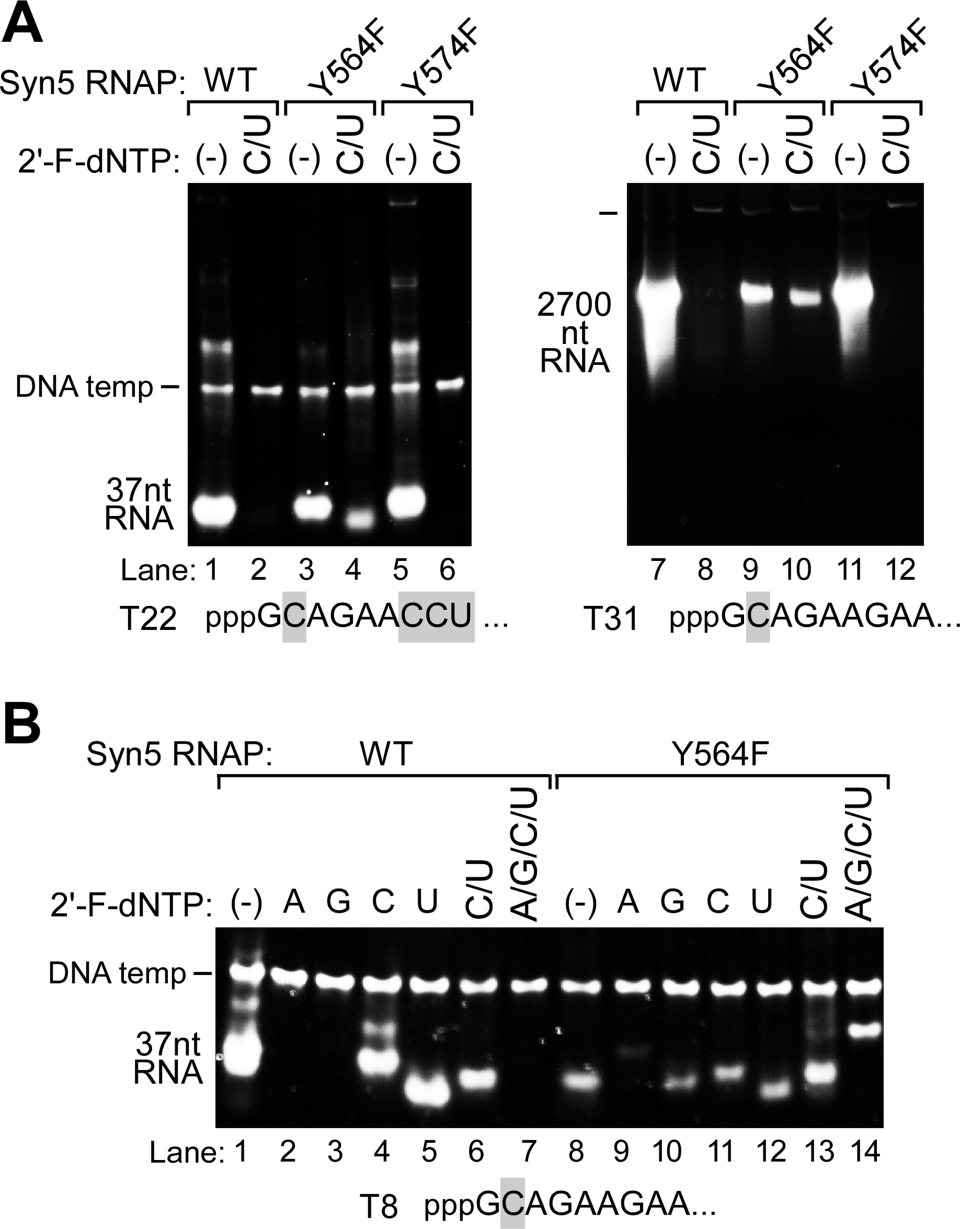
Incorporation of 2′-F-dNMPs by wild-type, Y564F and Y574F Syn5 RNA polymerases. Products of transcription reactions were separated by native gel electrophoresis and then stained with ethidium bromide**. (A)** Incorporation of 2′-F-dNMPs into 37 nt and 2700 nt transcripts using wild-type Syn5, Syn5-Y564F and Syn5-Y574F RNA polymerases. Transcription reactions were carried out using either template T22, which encodes a 37 nt transcript (lanes 1–6), or T31, which encodes a 2700 nt transcript (lanes 7–12). The sequence of the first nine nucleotides of each transcript is shown at the bottom of the gel, with the C and U residues in gray background. The RNA polymerase used for each reaction is indicated at the top of the gel. The reaction mixtures that contained only unmodified NTPs (‘-’) or both 2′-F-dCTP and 2′-F-dUTP (‘C/U’) are indicated at the top of the gel. Mg^2+^ was used as the only metal ion in all reactions. The position of the migration of the DNA templates and the RNA products are marked on the left of the gel. **(B)** Incorporation of 2′-F-dNMPs by Syn5 and Syn5-Y564F RNA polymerases. Transcription reactions were carried out using template T8, which encodes a 37 nt transcript. Reactions were carried out using either wild-type Syn5 RNA polymerase (lanes 1–6) or Syn5-Y564F RNA polymerase (lanes 7–14). Each of the four NTPs was individually replaced by the corresponding 2′-F analog, as indicated at the top of the gel. In the reactions carried out in lanes 6 and 12, both CTP and UTP were replaced by 2′-F-dCTP and 2′-F-dUTP. In the reactions carried out in lanes 7 and 14, all four NTPs were replaced by 2′-F-dNTPs. The position of the migration of the DNA templates and the RNA products are marked on the left of the gel.

**Figure 5.**
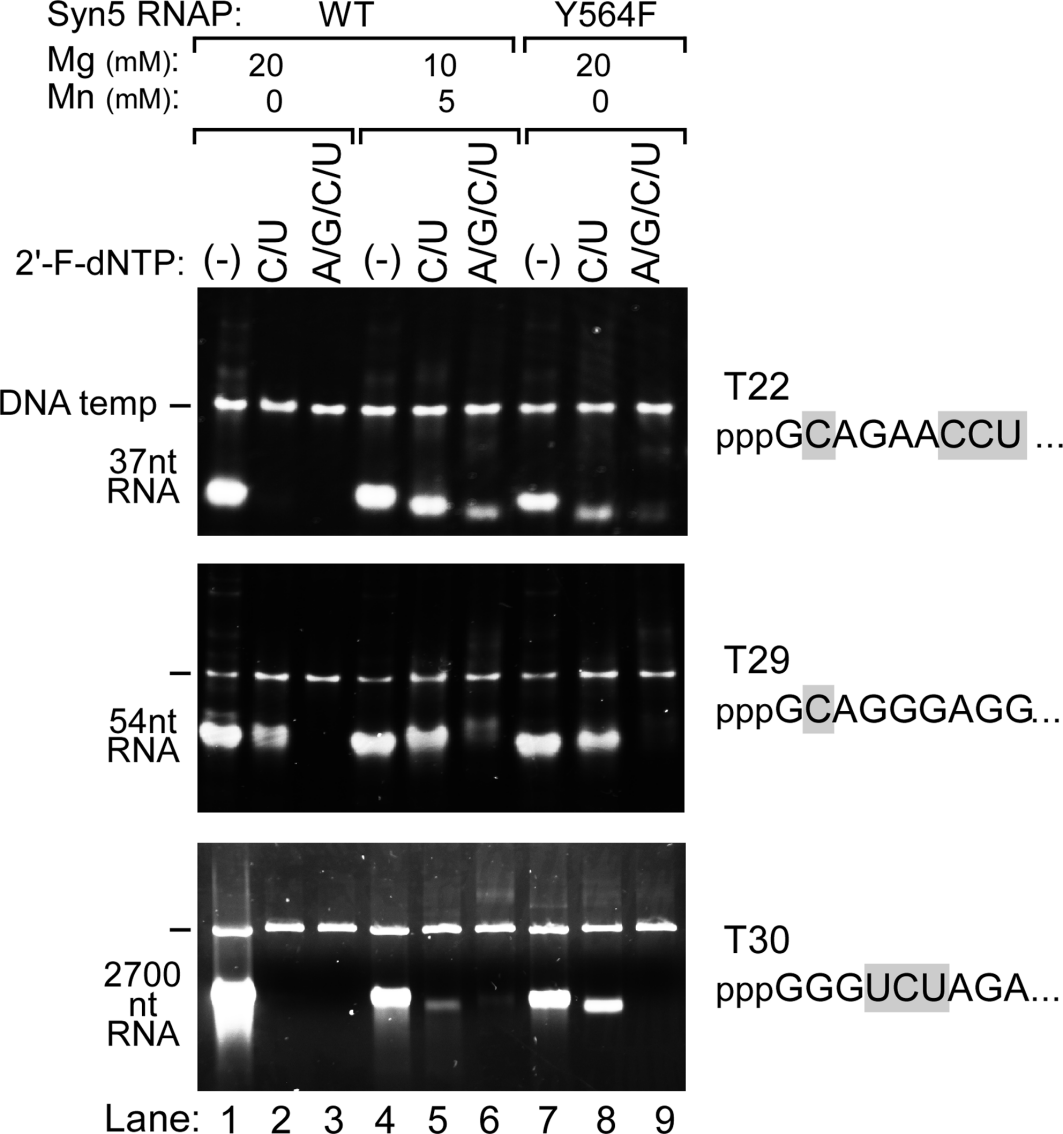
Synthesis of 2′-F RNAs by wild-type and Y564F Syn5 RNA polymerases on three different templates. Transcription reactions were carried out using either wild-type Syn5 RNA polymerase (lanes 1–6) or Syn5-Y564F RNA polymerase (lanes 7–9). Reactions were carried out in the presence of 20 mM Mg^2+^ (lanes 1–3 and 7–9) or a mixture of 10 mM Mg^2+^ and 5 mM Mn^2+^ (lanes 4–6). The reaction mixtures that contained only normal NTPs (lanes 1, 4 and 7), both 2′-F-dCTP and 2′-F-dUTP (lanes 2, 5 and 8), or all four 2′-F-dNTPs (lanes 3, 6 and 9) are shown at the top of the gel. Products of transcription reactions were separated by native gel electrophoresis and then stained with ethidium bromide. The position of the migration of the DNA templates and the RNA products are marked on the left of the gel. The template used for the top gel is T22, which encodes a 37 nt transcript. The template used for the middle gel is T29, which encodes a 54 nt transcript. The template used for the bottom gel is T30, which encodes a 2700 nt transcript. For each template, the sequence of the first nine nucleotides of the transcript is shown at the right of the gel, with the C and U residues in gray background.

**Figure 6.**
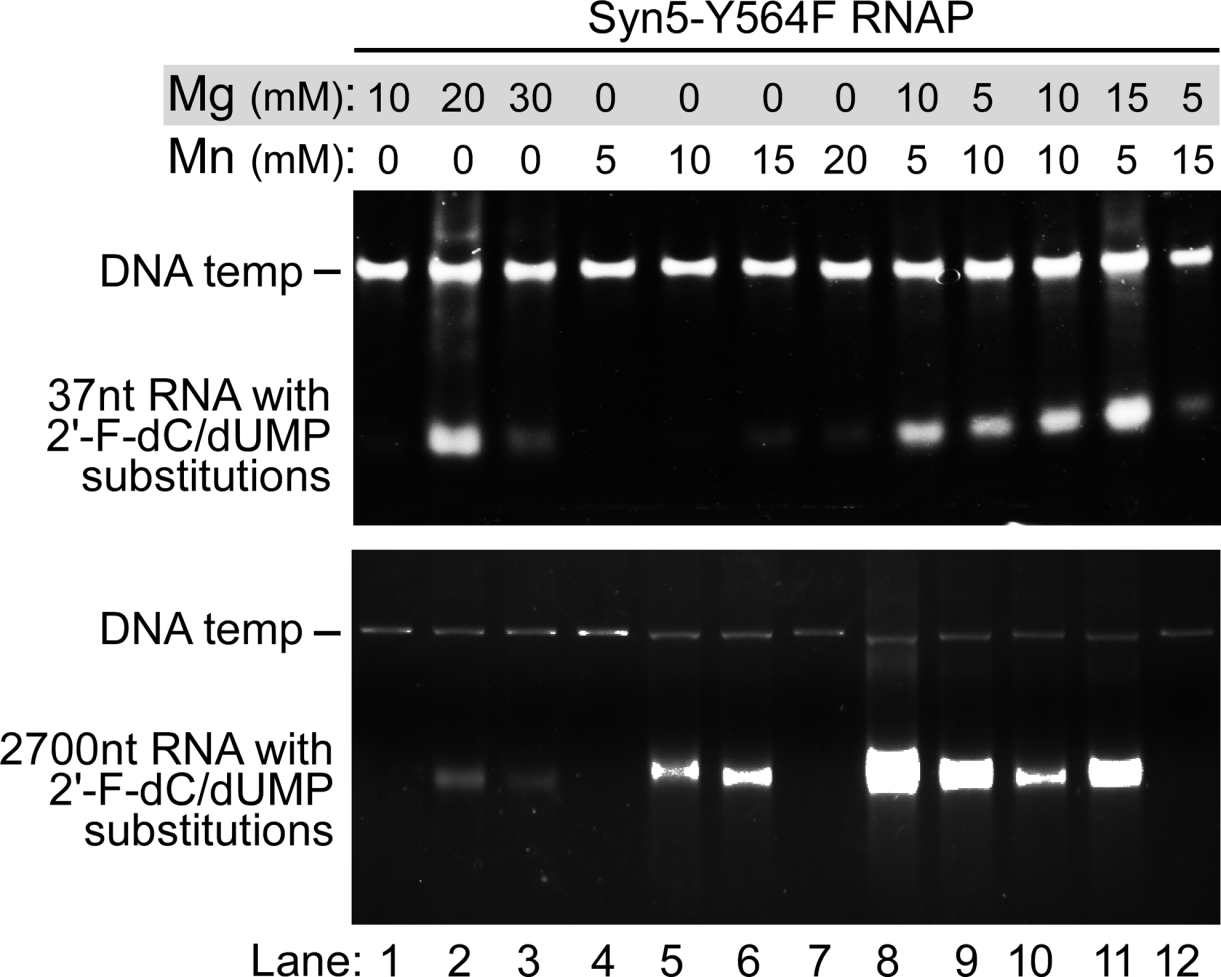
Effect of Mn^2+^ ions on 2′-F-dNMP incorporation by Syn5-Y564F RNA polymerase. Transcription reactions were carried out by Syn5-Y564F RNA polymerase. The metal ion present in each reaction and its concentration are shown at the top of the gel. All the reaction mixtures contained ATP, GTP, 2′-F-dCTP and 2′-F-dUTP. Products of transcription reactions were separated by native gel electrophoresis and then stained with ethidium bromide. The position of the migration of the DNA templates and the transcripts are marked. The template used for the top gel is T22, which encodes a 37 nt transcript. The template used for the bottom gel is T30, which encodes a 2700 nt transcript.

## RESULTS AND DISCUSSION

### Influence of initiation sequences on 2′-F-dNMP incorporation and transcription yield by Syn5 RNA polymerase

Currently, most of the applications for 2′-F RNA involve the incorporation of 2′-F-dCMP and 2′-F-dUMP into RNAs used for siRNA, RNA aptamers and ribozymes. Thus we initially focused on the incorporation of these analogs into small RNAs (<100 nt). We speculated that since cyanophage Syn5 is likely more ancient evolutionarily than bacteriophage T7, its enzymes may have more relaxed specificity for substrates. We initially examined the ability of wild-type Syn5 RNA polymerase to incorporate 2′-F-dNMPs into RNA. Syn5 RNA polymerase exhibits dramatically different abilities to incorporate each of the four different 2′-F-dNMPs into three different DNA templates that encode a 37 nt RNA, a 32 nt RNA and a tRNA (Figure 1A). In general, 2′-F-dCMP is incorporated much more efficiently than the other three analogs. Based on the sequences of these three templates we hypothesized that the efficiency of 2′-F-dNMPs incorporation by Syn5 RNA polymerase is strongly affected by the initiation sequence (i.e. the first 12 nucleotides) of the RNAs synthesized. It has been well documented in the literature that for T7 RNA polymerase, transcription initiation, or the synthesis of the first 12 nucleotides, is a more difficult process than elongation where the transcription complexes are considerably more stable (17). In fact, a large percentage of initiations by T7 RNA polymerase fail to successfully transition from the initiation complex to the transcription elongation stage, resulting in abortive products (17,18). It is therefore likely that the transcription initiation stage will be more sensitive to perturbations in the structure of the NTPs, such as the use of 2′-F analogs. On template T2, where there is only one C or U in the initiation region, 2′-F-dCMP or 2′-F-dUMP or both can be incorporated efficiently into the full-length transcript (Figure 1A, lanes 10–12). In contrast, on template T1, where all four bases are present multiple times adjacent to one another, none of the 2′-F analogs can be efficiently incorporated into the full-length transcript (Figure 1A, lanes 1–6). Template T3, that encodes one C in the initiation region as the initiating nucleotide, allows efficient incorporation of 2′-F-dCMP as the 5′ nucleotide (Figure 1A, lane 17). Although this template has multiple G's and U's, they are distal to the 5′ end, so that they have less of an inhibitory effect on the incorporation of 2′-F-dGMP and 2′-F-dUMP, respectively (Figure 1A, lanes 16 and 18). This effect will be discussed in more detail below.

To further test our hypothesis, we constructed another template (T4) that is the same as T1 except that C7C8U9U10 (numbers represent the position of the nucleotide in RNA counting from the 5′-end) was replaced by G7A8A9G10. We examined the ability of Syn5 RNA polymerase to initiate transcription on this template in the presence of 2′-F-dNTPs (Figure 1B). Template T4 has no C or U in the initiation region, resulting in very efficient incorporation of both 2′-F- dCMP and 2′-F-dUMP, with the total amount of transcript produced being at least 50% of that produced in the absence of any analogs (Figure 1B, compare lanes 1 and 4, 5 and 7; Supplementary Table S1). As expected, there is no transcription of this template in the presence of 2′-F-dATP (Figure 1B, lane 6).

When comparing T1 and T4 as templates for run-off transcription with Syn5 RNA polymerase, it is clear that not only the incorporation of 2′-F-dCMP and 2′-F-dUMP, but also the overall yield of transcripts in the absence of any analogs, are dramatically different. When C7C8U9U10 in T1 is replaced by G7A8A9G10 in T4, the yield increases 10-fold (Figure 1A, lane 1 versus Figure 1B, lane 1; Figure 2B, lane 1 versus lane 5; Supplementary Table S1). This difference was unexpected since previous studies with T7 RNA polymerase suggested that this region did not have a significant effect on transcription (19). In follow-up experiments, we carried out a more detailed investigation on the influence of the sequence in the 1–12 nt region sequence on the yield of transcription products using Syn5 RNA polymerase. We tested the effect of various sequences in this region using 28 different templates (Figure 2A and B). These templates are divided into four groups, each focusing on a different 3 nt region. Multiple product bands are observed in some lanes when the RNA is overloaded; reducing the amount of product loaded onto these gels results in bands with greater uniformity (for example, compare the sample loaded in Figure 2B, lane 1, with the identical sample loaded at half the concentration in Figure 2A, lane 1). It is likely that the multiple bands observed at high concentrations of RNA represent different secondary structures in the same RNA product.

The template region corresponding to nts 1–3 in the RNA is critical for transcription efficiency by T7 RNA polymerase (19). When sequences in this region are compared using Syn5RNA polymerase, we find that there is efficient transcription using a variety of sequences such as G1G2A3, G1A2A3, A1G2A3, G1C2A3 and C1A2G3 (Figure 2A, lanes 1 and 3–5; Figure 2B, lanes 5–7), with the highest yield observed using G1C2A3 (Figure 2A, lane 5 versus 1–4; Figure 2B, lane 6 versus 5 and 7). This result is consistent with the fact that in the cyanophage Syn5 genome the sequence G1C2A3 is found following both of the promoters (17). Surprisingly, the trinucleotide template sequence that provides for the greatest amount of transcription with T7 RNA polymerase (G1G2G3) supports the lowest yield by Syn5 RNA polymerase (Figure 2A, lane 2). We also examined transcription by T7 RNA polymerase on templates with various sequences at the first three nucleotide positions of the transcript. The results are consistent with previous results (19) that showed that G1G2G3 supports the highest yield (Figure 2B, lane 9), G1G2A3 and G1C2A3 work efficiently (Figure 2B, lanes 8 and 10), while with the template C1A2G3, transcription by T7 RNA polymerase is much less efficient compared to Syn5 RNA polymerase (Figure 2B, lane 11 versus 7).

For template sequences corresponding to the regions 4–6 and 7–9 of the transcript, we found that a consecutive stretch of three A's or U's in either of these regions of the transcript is very inhibitory to Syn5 RNA polymerase (Figure 2A, lanes 6 and 9, 7 and 11). However, upon inspection of the sequences of template T9 and T12, we found that these replacements result in a consecutive stretch of four (T9) and five (T12) A's in the initiation region, a scenario rare in most research applications. Since multiple and often consecutive A's are present in most templates that are transcribed efficiently, we believe that there must be more than three consecutive A's in the initiation region to be inhibitory to Syn5 RNA polymerase. Three consecutive U's in Templates T10 and T14 result in poor synthesis leading us to examine in greater detail the effect of multiple U's at different positions in the initiation region. When one U was introduced into the product of template T4 at position 5 (T19), the yield decreased about 3-fold (Figure 2B, lane 2 versus 1); an additional U in this region, whether at position 8 (T20) or 6 (T21), further decreased the yield by at least 20-fold (Figure 2B, lanes 3 and 4 versus 1). Other sequences, such as C7C8C9 and G7C8G9, also reduce the yield of transcripts produced by Syn5 RNA polymerase, but in these cases the amount of product was still substantial (Figure 2A, lanes 10 and 12). In the template region corresponding to nucleotides 10–12, all substitutions, including A10A11A12, U10U11U12 and C10C11C12, have minimal effect on the yield (Figure 2A, lanes 13–15 versus 1). We conclude from these data that more than two U's in the first nine nucleotides of the RNA product have a negative effect on transcription by Syn5 RNA polymerase. This conclusion is consistent with the sequence of genes transcribed from Syn5 RNA polymerase promoters; two such genes do not contain any U's in the initiation region of either transcript (20). Previous studies have shown that T7 RNA polymerase is unable to produce RNAs with a U at either the first or second position (21).

The plasmid template we used in our previous work (T30) was derived from plasmid pUC19 and encodes for an RNA transcript that starts with GGGUCUAGA (16). Based on the results just discussed, this sequence should be an inefficient template for Syn5 RNA polymerase, both because it starts with three consecutive G's, and there are two U's in the first nine nucleotides. We therefore constructed a template (T31) in which the sequence of the first nine nucleotides of the transcript, ‘GGGUCUAGA’, was replaced by the sequence ‘GCAGAAGAA’. The yield of transcripts synthesized by Syn5 RNA polymerase on this template is more than 100-fold greater than that produced using the original template, approaching ∼70 μg of RNA synthesized per 10 μl reaction (Figure 2B, compare lanes 14 and 13; Supplementary Table S1).

We have previously shown that transcription by Syn5 RNA polymerase that starts with U is very inefficient using 0.2 mM UTP (16). Increasing the concentration of UTP increases the yield of RNA produced by Syn5 RNA polymerase. When the reaction is carried out using 4 mM UTP, transcription is robust (Figure 2C, lane 1–3). Similarly, while transcription by Syn5 RNA polymerase that starts with GGG is very inefficient using 4 mM GTP, increasing the concentration of GTP to 8 mM significantly increases the amount of transcript synthesized (Figure 2C, lanes 4–9). In summary, with our current reaction conditions, there is no limitation on the sequence of the first three positions of the transcripts produced using Syn5 RNA polymerase. In contrast, increasing the concentration of UTP in the reaction mixture has little effect on improving the yield of RNAs that contain more than two U's in nucleotides 1–9 of the transcript (data not shown). Such templates should be avoided when designing transcription templates for use by Syn5 RNA polymerase.

Replacement of C7C8U9U10 by G7A8A9G10 increases the yield of transcripts produced by Syn5 RNA polymerase by a factor of 10 (Figure 2B, compare lanes 5 and 1). In contrast, this change only slightly increases the yield of transcripts using T7 RNA polymerase (Figure 2B, lane 12 versus 8). This result again demonstrates that each of these RNA polymerases have distinct sequence preferences for the RNA transcribed near the start of the transcripts.

Just as the sequence of the region corresponding to residues 10–12 of the transcript has little effect on the yield of transcripts produced by Syn5 RNA polymerase, the sequence of this region also has negligible effect on the incorporation of 2′-F-dNMPs (Figure 2D). On template T17, that encodes for an RNA transcript with U10U11U12, Syn5 RNA polymerase can efficiently incorporate 2′-F-dUMP, with at most a 3-fold reduction in the amount of transcripts (Figure 2D, compare lanes 1 and 2). Transcription by Syn5 RNA polymerase on template T18, which encodes an RNA transcript with C10C11C12, the yield of transcripts is comparable using either CTP or 2′-F-dCTP (Figure 2D, lanes 3 and 4). In summary, the efficiency of incorporation of 2′-F-dUMP and 2′-F-dCMP into transcripts using Syn5 RNA polymerase will depend on the sequence of the first nine nucleotides of the transcript: if the RNA transcript has less than two C's or U's in the first nine nucleotides, then Syn5 RNA polymerase will efficiently incorporate these analogs using the standard conditions described here.

We determined whether the above rules would also be applicable to the incorporation of 2′-F-dCMP and 2′-F-dUMP using T7 RNA polymerase. We constructed DNA template T28 that encodes the same RNA as that encoded by template T4 for Syn5 RNA polymerase (lacking any C's or U's in the first nine nucleotides). On this template, 2′-F-dCTP and 2′-F-dUTP inhibit the amount of transcripts produced by T7 RNA polymerase, at least by a factor of 10 (Figure 2E, compare lanes 2 and 1). This result is in dramatic contrast to the minimal effect that these analogs have on transcription by Syn5 RNA polymerase (Figure 2E, compare lanes 4 and 3).

### Effect of manganese ions on the incorporation of 2′-F-dNMPs by Syn5 RNA polymerase

Templates encoding multiple C's and U's in the initiation region (nucleotides 1–9) are not transcribed efficiently by Syn5 RNA polymerase in the presence of 2′-F-dCTP and 2′-F-dUTP using the standard conditions described above. We varied reaction conditions in order to optimize transcription on these templates. Manganese ions decrease the substrate discrimination for many polymerases (22). T7 RNA polymerase can use Mn^2+^ instead of Mg^2+^ for catalysis; the optimum concentration of Mn^2+^ is 2.0–2.5 mM (23), 10-fold lower than the optimum concentration of Mg^2+^. However, even at the optimum metal concentrations, the activity of T7 RNA polymerase with Mn^2+^ is much lower than that with Mg^2+^ (13). A mixture of Mn^2+^ and Mg^2+^ ions has been used successfully (23,24) to provide the two metal ions required for catalysis in the T7 RNA polymerase active site (17). Presumably, some T7 RNA polymerase molecules will have one Mn^2+^ and one Mg^2+^ in their active sites, resulting in relaxed specificity for substrate analogs. However, since concentrations of Mn^2+^ ions greater than 2.5 mM are highly inhibitory to T7 RNA polymerase, the concentration of Mg^2+^ ions in these mixtures must also be reduced to levels that are suboptimal for T7 RNA polymerase activity.

The natural hosts of cyanophage Syn5 are ocean-dwelling cyanobacteria. In this environment, the intracellular concentration of Mn^2+^ is 100 times the concentration found in *E. coli*, the natural host for bacteriophage T7 (25,26). Thus, it is logical to speculate that Syn5 RNA polymerase will retain activity at higher Mn^2+^ concentrations than will T7 RNA polymerase. Indeed, under standard reaction conditions, we find that the optimum Mn^2+^ concentration for Syn5 RNA polymerase activity is 15–20 mM (Figure 3A), similar to its optimum Mg^2+^ concentration. The yield of transcripts produced in the presence of the optimum concentration of Mn^2+^ is several-fold lower than that produced at the optimum concentration of Mg^2+^ (Figure 3A, lane 12 versus lane 2). However, the incorporation of 2′-F-dCMP and 2′-F-dUMP by Syn5 RNA polymerase is more efficient in the presence of 15 mM Mn^2+^ than in the presence of 20 mM Mg^2+^ (Figure 3A, lane 11 versus lane 3). Since the optimal concentrations for each metal ion in Syn5 transcription reactions are similar, both ions can be present together in the Syn5 transcription reaction at high concentrations. With the combination of 10 mM Mg^2+^ and 5 mM Mn^2+^, the yield of transcripts produced using all natural NTPs is within 50% of that observed in the presence of only 20 mM Mg^2+^ (Figure 3B, compare lanes 5 and lane 1). Under these conditions, using a mixture of the two metal ions, the incorporation of 2′-F-dCMP and 2′-F-dUMP is substantially improved compared to reactions that contain only 15 mM Mn^2+^ (Figure 3B, compare lanes 6 and 4). Thus this combination of the two metal ions provides the best balance between a high yield of transcripts and low discrimination against 2′-F-dCTP and 2′-F-dUTP.

In the presence of all four 2′-F-dNTPs, Syn5 RNA polymerase synthesizes a significant amount of products containing the complete substitution of 2′-fluoro analogs, either in the presence of 10 mM Mg^2+^ and 5 mM Mn^2+^ or 5 mM Mg^2+^ and 10 mM Mn^2+^ (Figure 3B, lanes 7 and 10). The highest amount of transcripts containing all four 2′-F-dNMPs are observed when the first three nucleotides of the product are the preferred sequence GCA (template T22 in Figure 3C) and the metal mixture consists of 10 mM Mg^2+^ and 5 mM Mn^2+^. Under these conditions, the yield of product containing all four 2′-F-dNMPs is about 10-fold lower than that produced using natural NTPs (Figure 3C, compare lanes 6 and 4) and 4-fold lower than transcripts containing only 2′-F-dCMP and 2′-F-dUMP analogs (Figure 3C, compare lanes 5 and 4). In these examples, the templates contains multiple C's in the first nine nucleotides of the transcript, so under standard reaction conditions the Syn5 RNA polymerase does not yield any product (Figure 3B lane 2; Figure 3C lanes 2 and 3). The use of a high concentration of both Mg^2+^ and Mn^2+^ improves dramatically the yield of transcripts produced by wild-type Syn5 RNA polymerase with 2′-F-dNTPs.

### Effect of Y564F and Y574F mutations on 2′-F-dNMP incorporation by Syn5 RNA polymerase

Although Syn5 RNA polymerase efficiently produces small 2′-F RNAs (2′-F DNAs) in the presence of a high concentration of Mg^2+^ and Mn^2+^, the synthesis of long 2′-F RNAs is poor (Figure 3C). For example, Syn5 RNA polymerase synthesizes a large amount of a 2700 nt RNA with the four natural rNTPs (Figure 3C, lane 7), whereas the amount of product synthesized when 2′-F-dCTP and 2′-F-dUTP are substituted for CTP and UTP is barely detectable (Figure 3C, lane 8). In order to improve the efficiency of the production of long 2′-F RNAs, we analyzed the effect of genetic alterations in the Syn5 RNA polymerase. A T7 RNA polymerase with the tyrosine at residue 639 replaced by phenylalanine (Y639F) improves the efficiency with which T7 RNA polymerase incorporates nucleotide analogs (13). We analyzed the amino acid sequence of Syn5 RNA polymerase to locate the residue homologous to Y639 of T7 RNA polymerase. The amino acid sequence homology between these two enzymes is less than 30% and the region around Y639 of T7 RNA polymerase is not well conserved. We identified two tyrosine residues, Y564 and Y574 of Syn5 RNA polymerase in a location that potentially could alter the recognition of the nucleoside 2′-group by the polymerase (Supplementary Figure S2A). We constructed two Syn5 RNA polymerases that harbor either a Y564F (Syn5-Y564 RNA polymerase) or Y574F (Syn5-Y574F RNA polymerase) alteration, and purified each of the overproduced enzymes to homogeneity, using the procedures described for the wild-type Syn5 RNA polymerase. In both cases we obtained the same high yield as that obtained for the wild-type protein (Supplementary Figure S2B).

We examined the ability of the two altered Syn5 RNA polymerases to synthesize transcripts containing 2′-F-dCMP and 2′-F-dUMP. Syn5-Y574F RNA polymerase behaves similar to wild-type Syn5 RNA polymerase with regard to the ability to incorporate 2′-F-dCMP and 2′-F-dUMP (Figure 4A lanes 1, 2, 5–8, 11 and 12). Syn5-Y564F RNA polymerase, on the other hand, synthesizes 2′-F-RNA much more efficiently than does wild-type Syn5 RNA polymerase. In the presence of 2′-F-dCTP and 2′-F-dUTP, the amount of transcript produced is 30% that observed with CTP and UTP on templates producing both a 37 nt RNA and a 2700 nt RNA (Figure 4A lanes 3, 4, 9 and 10). The overall yield of RNA produced with this mutant RNA polymerase is reduced compared to wild-type Syn5 RNA polymerase (Figure 4A lane 3 versus 1 and lane 9 versus 7). Nevertheless, the yield of RNA's containing 2′-F-dCMP and 2′F-dUMP produced by Syn5-Y564F RNA polymerase can reach 0.4 μg/μl reaction for a 37 nt RNA and 1 μg/μl reaction for a 2700 nt RNA (Figure 4A, lanes 4 and 10).

On a template encoding an RNA lacking U and with only one C in the initiation region (T8), wild-type Syn5 RNA polymerase efficiently incorporates 2′-F-dCMP and 2′-F-dUMP (Figure 4B, lanes 4–6), while no incorporation is observed using 2′-F-dATP or 2′-F-dGTP (Figure 4B, lanes 2 and 3). Syn5-Y564F RNA polymerase, however, can synthesize transcripts that contain all four 2′-F-dNMPs (Figure 4B, lane 9–12), albeit with varying efficiencies. Using this template, the Syn5-Y564F RNA polymerase synthesizes a 37 nt RNA with complete replacement of NMPs by 2′-F-dNMPs at a yield of 0.1 μg/μl reaction (Figure 4B, lane 14).

### Synthesis of 2′-F RNAs (2′-F DNAs) with Syn5 RNA polymerases

We compared the three methods summarized above (I. wild-type Syn5 RNA polymerase with Mg^2+^ alone; II. wild-type Syn5 RNA polymerase with a mixture of Mg^2+^ and Mn^2+^; and III. Syn5-Y564F RNA polymerase with Mg^2+^ alone) for their efficiency in transcribing different templates (Figure 5). For the incorporation of 2′-F-dCMP and 2′-F-dUMP, wild-type Syn5 RNA polymerase with a mixture of Mg^2+^ and Mn^2+^ and Syn5-Y564F RNA polymerase with Mg^2+^ alone are effective at producing a 37 nt RNA and a 54 nt RNA (Figure 5 top and middle gels, lanes 5 and 8). However, wild-type Syn5 RNA polymerase with a mixture of Mg^2+^ and Mn^2+^ results in a superior yield (>0.8 μg/μl reaction). With Mg^2+^, Mn^2+^ and wild-type Syn5 RNA polymerase, all four rNMPs can be substituted with 2′-F-dNMPs at a yield of ∼0.1 μg/μl reaction. The use of wild-type Syn5 RNA polymerase with just Mg^2+^ ions is effective only when the small RNA product has no U and one C in the first nine nucleotides of the transcript (Figure 5 middle gel, lane 2); however, this standard reaction mixture does provide a simple and robust method of transcription of such small RNAs containing 2′-fluoro substitutions.

For the production of long 2′-F RNAs, Syn5-Y564F RNA polymerase is the most efficient enzyme on templates that contain an optimized initiation region (Figure 4A, lane 10 versus Figure 3C, lane 8). We also examined Syn5-Y564F RNA polymerase on a very difficult template that produces a transcript starting with GGG and contains two U's in the first nine nucleotides of the transcript (template T30). Although the yield on this template is low (Figure 2B lane 13), a prolonged incubation to 8 h significantly increases the yield (Figure 5 bottom gel, lane 1), allowing us to compare various methods to optimize the amount of product synthesized. Again Syn5-Y564F RNA polymerase was superior to wild-type Syn5 RNA polymerase for the incorporation of 2′-F-dCMP and 2′-F-dUMP (Figure 5 bottom gel, lane 8 versus lanes 5 and 2). However, for the incorporation of all four 2′-F-dNMPs into the transcript produced on this template, the only conditions that produced any full-length product were those using wild-type Syn5 RNA polymerase with a mixture of Mg^2+^ and Mn^2+^ ions. Even under these conditions the efficiency was very low (Figure 5 bottom gel, lane 6).

We tested the effect of Mn^2+^ on the 2′-F RNA synthesis catalyzed by Syn5-Y564F RNA polymerase and found that the addition of Mn^2+^ did not improve the yield of a small 2′-F RNA (37 nt) with 2′-F-dCMP and 2′-F-dUMP substitutions as it does for wild-type enzyme (Figure 3A) at all conditions tested (Figure 6 upper gel, lanes 4–12 versus 2). However, in many reactions containing Mn^2+^, the yield of a long 2′-F RNA (2700 nt) with 2′-F-dCMP and 2′-F-dUMP substitutions is significantly improved compared to that with Mg^2+^ only (Figure 6 bottom gel, lanes 5, 6, 8–11 versus 2). The highest yield was observed with a combination of 10 mM Mg^2+^ and 5 mM Mn^2+^ (Figure 6 bottom gel, lane 8). However, the yield of 2′-F DNA with full 2′-F-dNMP substitutions by Syn5-Y564F RNA polymerase with any combination of Mg^2+^ and Mn^2+^ tested is still too low to be detected (Supplementary Figure S3).

**Figure 7.**
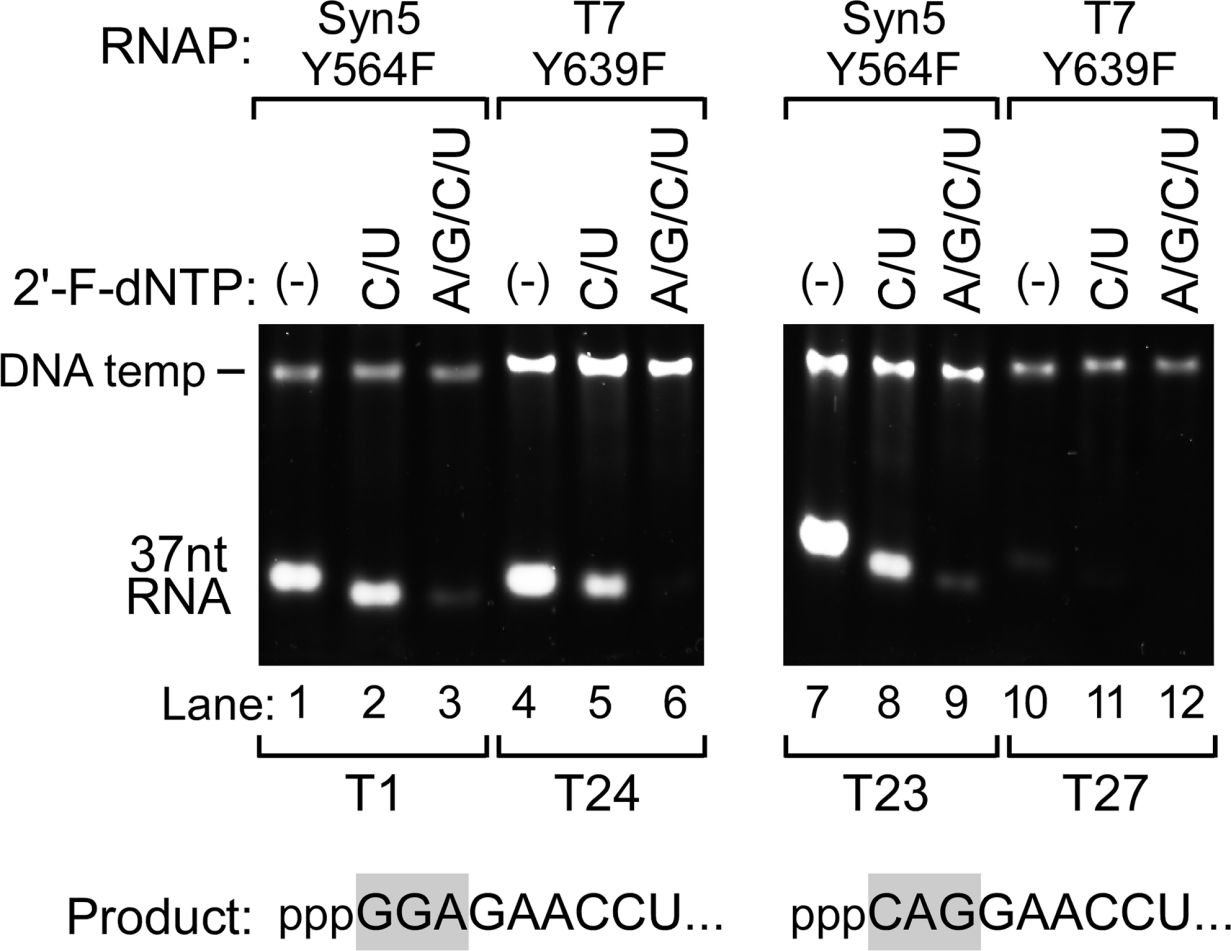
Synthesis of 2′-F RNAs by Syn5-Y564F and T7-Y639F RNA polymerases. Transcription reactions were carried out using either Syn5-Y564F RNA polymerase (lanes 1–3 and 7–9) or T7-Y639F RNA polymerase (lanes 4–6 and 10–12). The reaction mixtures that contained only normal NTPs (lanes 1, 4, 7 and 10), both 2′-F-dCTP and 2′-F-dUTP (lanes 2, 5, 8 and 11), or all four 2′-F-dNTPs (lanes 3, 6, 9 and 12) are shown at the top of the gel. Products of transcription reactions were separated by native gel electrophoresis and then stained with ethidium bromide. The position of the migration of the DNA templates and the RNA products are marked on the left of the gel. The template used for the experiments shown in the left gel is T1 (for Syn5-Y564F RNA polymerase) and T24 (for T7-Y639F RNA polymerase), which encode 37 nt transcripts of the same sequence starting with GGA. The template used for the right gel is T23 (for Syn5-Y564F RNA polymerase) and T27 (for T7-Y639F RNA polymerase), which encode 37 nt transcripts of the same sequence starting with CAG. For each template, the sequence of the first nine nucleotides of the transcript is shown at the bottom of the gel, with the first three residues in a gray background.

Finally we compared the Syn5-Y564F RNA polymerase to T7-Y639F RNA polymerase, the standard tool for synthesis of 2′-F RNA (2′-F DNA), with various initial sequences in the template (Figure 7). When the transcripts start with GGA, both enzymes efficiently produce 2′-F RNA with 2′-F-dCMP and 2′-F-dUMP substitutions (Figure 7, lanes 2 and 5). Although the yield of natural RNA is similar for both enzymes (Figure 7, lane 1 versus 4), the yield of 2′-F RNA with 2′-F-dCMP and 2′-F-dUMP substitutions synthesized by T7-Y639F RNA polymerase is lower than that synthesized by Syn5-Y564F RNA polymerase (Figure 7, lane 2 versus 5). 2′-F DNA with full substitutions synthesized by Syn5-Y564F RNA polymerase (Figure 7, lane 3) is not observed with T7-Y639F RNA polymerase (Figure 7, lane 6), indicating that T7-Y639F RNA polymerase has stronger discrimination against 2′-F-dNTPs, especially 2′-F-dGTP and/or 2′-F-dATP than does Syn5-Y564F RNA polymerase. When the transcripts start with CAG, Syn5-Y564F RNA polymerase showed a similar yield with that for transcripts starting with GGA (Figure 7, lanes 7–9). In the latter case the products synthesized by T7-Y639F RNA polymerase, even natural RNA, are barely detectable. Syn5-Y564F RNA polymerase is advantageous to synthesize 2′-F RNA (2′-F DNA) that is heavily modified and to synthesize transcripts containing initial sequences that are not synthesized effectively by T7 RNA polymerase.

## SUPPLEMENTARY DATA

Supplementary Data are available at NAR Online.

SUPPLEMENTARY DATA
