# Supplementary material for: Synthesis of 2′-Fluoro RNA by Syn5 RNA polymerase
Source: Nucleic Acids Res. 2015 Apr 20;43(14):e94. doi: 10.1093/nar/gkv367 (PMC4538805; doi:10.1093/nar/gkv367)
Supplement: SUPPLEMENTARY DATA [file supp_43_14_e94__index.html]

Synthesis of 2′-Fluoro RNA by Syn5 RNA polymerase — Synthesis of 2′-Fluoro RNA by Syn5 RNA polymerase — SUPPLEMENTARY DATA 

# Synthesis of 2′-Fluoro RNA by Syn5 RNA polymerase

## SUPPLEMENTARY DATA

**Files in this Data Supplement:**

- SUPPLEMENTARY DATA
