# Supplementary material for: Synthesis of 2′-Fluoro RNA by Syn5 RNA polymerase
Source: Nucleic Acids Res. 2015 Apr 20;43(14):e94. doi: 10.1093/nar/gkv367 (PMC4538805; doi:10.1093/nar/gkv367)
Supplement: SUPPLEMENTARY DATA [file supp_gkv367_nar-03725-met-g-2014-File009.pdf]

**Supplementary Table 1.**

**Table S1.** Transcription product yield of reactions described in this work.

| Reaction (10 μl) corresponding to | | Product yield (μg) |
|---|---|---|
| **Figure** | 1A | |
| **Lane** | 1 | 4.5 |
| | 2-3 | n.d.[a] |
| | 4 | 0.1 |
| | 5 | 0.2 |
| | 6 | n.d. |
| | 7 | 2.5 |
| | 8, 9 | n.d. |
| | 10 | 1.1 |
| | 11 | 0.4 |
| | 12 | 1.0 |
| | 13 | n.d. |
| | 14 | 2.2 |
| | 15 | n.d. |
| | 16 | 0.8 |
| | 17 | 1.8 |
| | 18 | 0.4 |
| | 19 | 0.2 |
| | 20 | n.d. |
| **Figure** | 1B | |
| **Lane** | 1 | 46 |
| | 2 | 38 |
| | 3 | 32 |
| | 4 | 23 |
| | 5 | 45 |
| | 6 | n.d. |
| | 7 | 23 |
| **Figure** | 2A | |
| **Lane** | 1 | 39 |
| | 2 | 2.1 |
| | 3 | 22 |
| | 4 | 14 |
| | 5 | 48 |
| | 6 | n.d. |
| | 7 | n.d. |
| | 8 | 48 |
| | 9 | n.d. |
| | 10 | 6.2 |
| | 11 | 0.1 |
| | 12 | 6.8 |
| | 13 | 30 |
| | 14 | 36 |

| | | |
|---|---|---|
| | 15 | 24 |
| **Figure** | 2B | |
| **Lane** | 1 | 50 |
| | 2 | 17 |
| | 3 | 1.7 |
| | 4 | 1.8 |
| | 5 | 6.2 |
| | 6 | 12 |
| | 7 | 7.6 |
| | 8 | 8.0 |
| | 9 | 14 |
| | 10 | 12 |
| | 11 | 1.1 |
| | 12 | 13 |
| | 13 | 0.6 |
| | 14 | 70[b] |
| **Figure** | 2C | |
| **Lane** | 1 | 1.5 |
| | 2 | 5.5 |
| | 3 | 11 |
| | 4 | 4.0 |
| | 5 | 9.3 |
| | 6 | 13 |
| | 7 | 1.3 |
| | 8 | 2.5 |
| | 9 | 3.7 |
| **Figure** | 2D | |
| **Lane** | 1 | 42 |
| | 2 | 15 |
| | 3 | 22 |
| | 4 | 20 |
| **Figure** | 2E | |
| **Lane** | 1 | 14 |
| | 2 | 1.1 |
| | 3 | 45 |
| | 4 | 23 |
| **Figure** | 3A | |
| **Lane** | 1 | - |
| | 2 | 6.1 |
| | 3-5 | n.d. |
| | 6 | 0.3 |
| | 7 | 0.1 |
| | 8 | 0.7 |
| | 9 | 0.1 |
| | 10 | 1.4 |
| | 11 | 0.4 |

| | | |
|---|---|---|
| | 12 | 1.7 |
| | 13 | 0.3 |
| **Figure** | 3B | |
| **Lane** | 1 | 5.7 |
| | 2 | n.d. |
| | 3 | 1.6 |
| | 4 | 0.3 |
| | 5 | 3.9 |
| | 6 | 0.8 |
| | 7 | 0.5 |
| | 8 | 2.7 |
| | 9 | 0.3 |
| | 10 | 0.5 |
| | 11 | 2.1 |
| | 12 | 0.1 |
| | 13 | 0.1 |
| **Figure** | 3C | |
| **Lane** | 1 | 17 |
| | 2, 3 | n.d. |
| | 4 | 12 |
| | 5 | 6.1 |
| | 6 | 1.3 |
| | 7 | 70 |
| | 8 | n.d. |
| **Figure** | 4A | |
| **Lane** | 1 | 15 |
| | 2 | n.d. |
| | 3 | 11 |
| | 4 | 4.0 |
| | 5 | 14 |
| | 6 | n.d. |
| | 7 | 70 |
| | 8 | n.d. |
| | 9 | 28 |
| | 10 | 10 [b] |
| | 11 | 62 |
| | 12 | n.d. |
| **Figure** | 4B | |
| **Lane** | 1 | 22 |
| | 2, 3 | n.d. |
| | 4 | 13 |
| | 5 | 12 |
| | 6 | 4.7 |
| | 7 | n.d. |
| | 8 | 2.4 |
| | 9 | 0.1 |

| | | |
|---|---|---|
| | 10 | 0.4 |
| | 11 | 0.8 |
| | 12 | 0.9 |
| | 13 | 2.7 |
| | 14 | 1.4 |
| **Figure** | 5 top gel | |
| **Lane** | 1 | 16 |
| | 2, 3 | n.d. |
| | 4 | 14 |
| | 5 | 8.0 |
| | 6 | 1.5 |
| | 7 | 11 |
| | 8 | 2.8 |
| | 9 | 0.5 |
| **Figure** | 5 middle gel | |
| **Lane** | 1 | 18 |
| | 2 | 8.8 |
| | 3 | n.d. |
| | 4 | 17 [b] |
| | 5 | 10 [b] |
| | 6 | 1.2 [b] |
| | 7 | 16 |
| | 8 | 8 |
| | 9 | 0.2 |
| **Figure** | 5 bottom gel | |
| **Lane** | 1 | 10 |
| | 2, 3 | n.d. |
| | 4 | 2.5 |
| | 5 | 0.2 |
| | 6 | n.d. |
| | 7 | 2.0 |
| | 8 | 0.9 |
| | 9 | n.d. |
| **Figure** | 6 top gel | |
| **Lane** | 1 | n.d. |
| | 2 | 3.5 |
| | 3 | 0.4 |
| | 4, 5 | n.d. |
| | 6 | 0.1 |
| | 7 | 0.2 |
| | 8 | 1.0 |
| | 9 | 0.7 |
| | 10 | 1.2 |
| | 11 | 3.4 |
| | 12 | 0.4 |
| **Figure** | 6 bottom gel | |

| | | |
|---|---|---|
| **Lane** | 1 | n.d. |
| | 2 | 0.8 |
| | 3 | 0.2 |
| | 4 | n.d. |
| | 5 | 2.5 |
| | 6 | 3.1 |
| | 7 | n.d. |
| | 8 | 20 [b] |
| | 9 | 11 |
| | 10 | 2.6 |
| | 11 | 9.8 |
| | 12 | n.d. |
| **Figure** | 7 | |
| **Lane** | 1 | 6.1 |
| | 2 | 3.9 |
| | 3 | 0.6 |
| | 4 | 7.5 |
| | 5 | 1.9 |
| | 6 | n.d. |
| | 7 | 6.6 |
| | 8 | 3.2 |
| | 9 | 0.7 |
| | 10 | 0.7 |
| | 11, 12 | n.d. |

Transcription yield was calculated based on comparison of the intensity of the product band to that of the DNA template band.

[a]n.d., not detected.

[b]Yield quantified by measurement of the purified transcript (see Materials and Methods).

**Supplementary Figure 1**

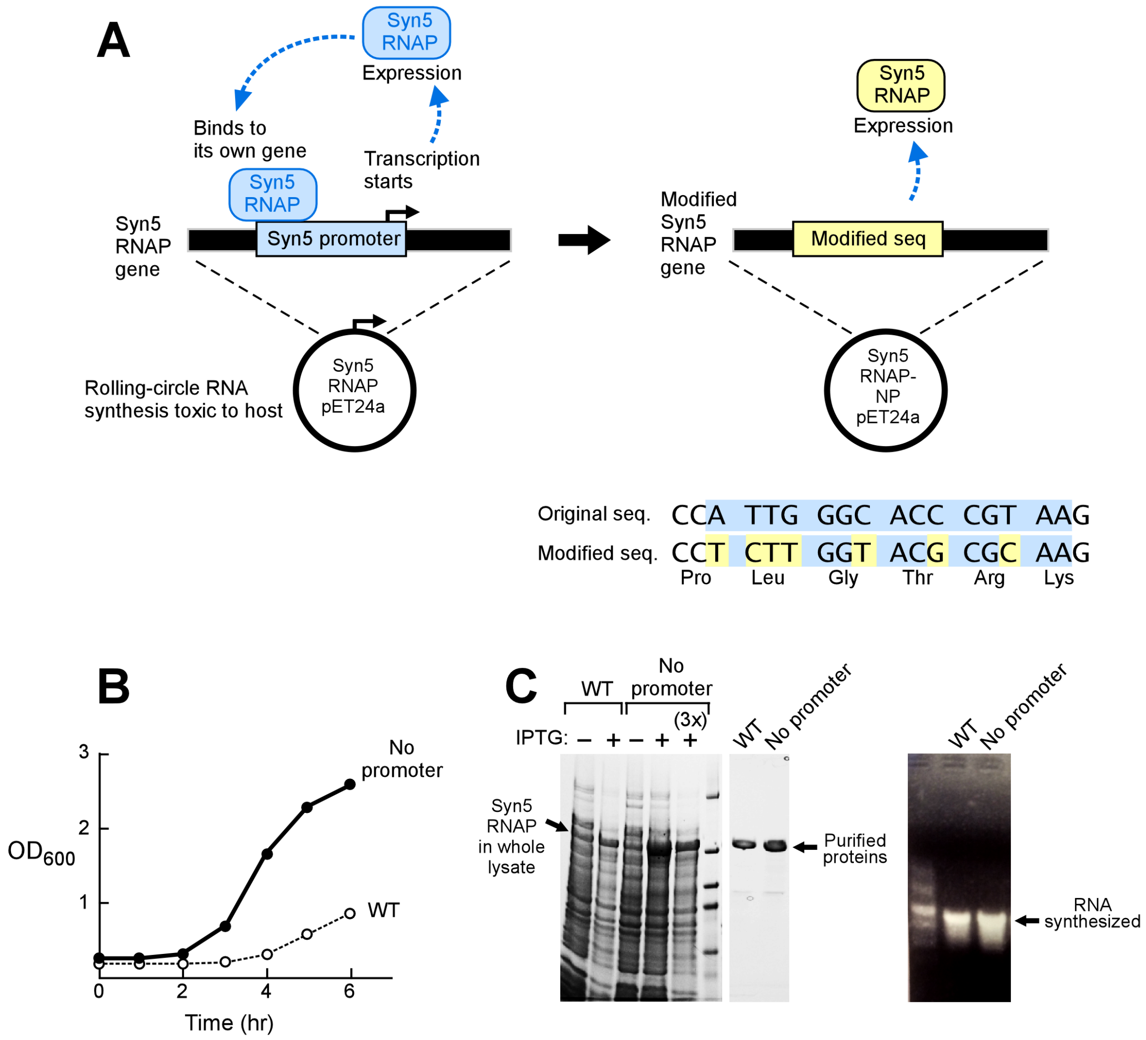


**Figure S1.** Modified Syn5 RNA polymerase gene for improved expression in *E. coli* cells. **(A)** The internal Syn5 promoter in the Syn5 RNA polymerase gene was modified without changing the encoded amino acids, in order to abolish rolling-circle RNA synthesis that will deplete the NTP pool in *E. coli* cells when Syn5 RNA polymerase is expressed. **(B)** *E. coli* cells harboring the modified vector grow much faster than those harboring the original vectors. **(C)** The modified vector results in higher expression of Syn5 RNA polymerase without affecting its enzyme activity.

**Supplementary Figure 2**

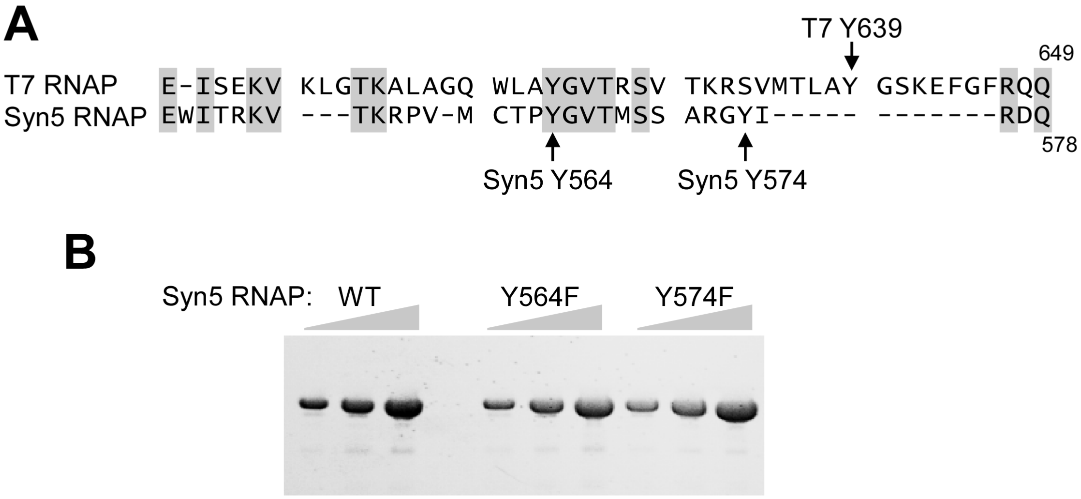


**Figure S2.** Syn5-Y564F and Syn5-Y574F RNA polymerases. **(A)** Syn5-Y564F and Syn5-Y574F RNA polymerases were constructed based on sequence alignment (using CLC Sequence Viewer 6) to the region containing Y639 in T7 RNA polymerase. **(B)** Both mutant enzymes were purified to homogeneity and analyzed by SDS-PAGE gel. The purified wild-type Syn5 RNA polymerase is shown on the left for comparison. Three concentrations (1, 2 and 4 μg) of each RNA polymerase were loaded into adjacent wells.

**Supplementary Figure 3**

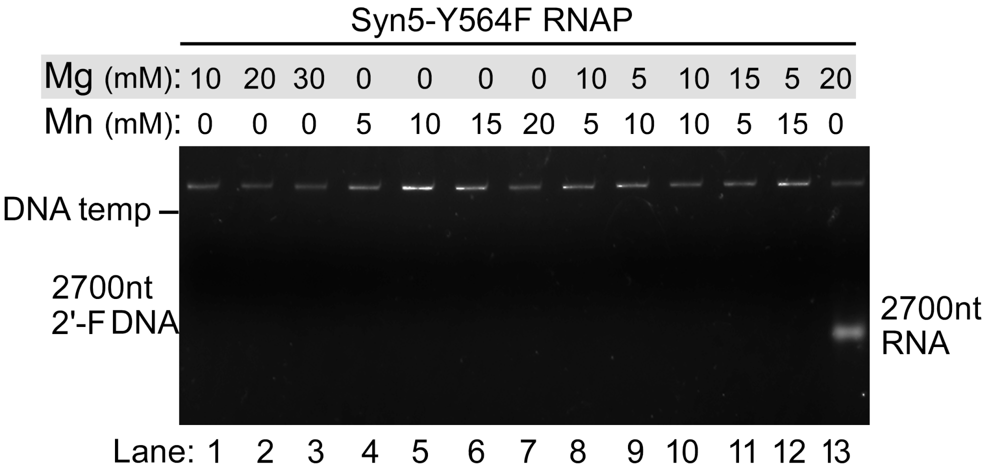


**Figure S3.** Effect of $Mn^{2+}$ ions on 2'-F DNA synthesis by Syn5-Y564F RNA polymerase. Transcription reactions were carried out by Syn5-Y564F RNA polymerase. The metal ion present in each reaction and its concentration are shown at the top of the gel. All the reaction mixtures contained four 2'-F-dNTPs except for lane 13, which contained four rNTPs. Products of transcription reactions were separated by native gel electrophoresis and then stained with ethidium bromide**.** The position of the migration of the DNA templates and the transcripts are marked. The DNA template used is T30, which encodes a 2,700 nt transcript.
